# Biomaterials Based on Chitosan and Its Derivatives and Their Potential in Tissue Engineering and Other Biomedical Applications—A Review

**DOI:** 10.3390/molecules28010247

**Published:** 2022-12-28

**Authors:** Marta Szulc, Katarzyna Lewandowska

**Affiliations:** Department of Biomaterials and Cosmetic Chemistry, Faculty of Chemistry, Nicolaus Copernicus University in Toruń, Gagarin 7, 87-100 Torun, Poland

**Keywords:** chitosan, chitosan derivatives, cross-linking, biomaterials, polysaccharides

## Abstract

In the times of dynamically developing regenerative medicine, more and more attention is focused on the use of natural polymers. This is due to their high biocompatibility and biodegradability without the production of toxic compounds, which means that they do not hurt humans and the natural environment. Chitosan and its derivatives are polymers made most often from the shells of crustaceans and are biodegradable and biocompatible. Some of them have antibacterial or metal-chelating properties. This review article presents the development of biomaterials based on chitosan and its derivatives used in regenerative medicine, such as a dressing or graft of soft tissues or bones. Various examples of preparations based on chitosan and its derivatives in the form of gels, films, and 3D structures and crosslinking products with another polymer are discussed herein. This article summarizes the latest advances in medicine with the use of biomaterials based on chitosan and its derivatives and provides perspectives on future research activities.

## 1. Introduction

Tissue engineering is focused on the creation of tissues to repair or replace diseased or damaged organs. Recently, natural polymers have become of increasing interest due to rapidly developing medical applications. This is due to their biodegradability and non-toxicity. They also mimic tissue structure better than synthetic polymers due to their physicochemical similarity. The development of new products based on tissue-mimicking biopolymers that are more robust, non-toxic, and biodegradable is a key issue that will guarantee rapid growth in the development of tissue engineering. Biomimetic natural polymers and hybrid polymer materials have the advantage of combining desired functions with tailored morphology and superior chemical and physical stability. These polymeric materials aim to cover all aspects of the subject, including, for instance, the design of hybrid materials, films, gel, sponge, nanocomposites, and hydrogels, without forgetting studies of structure–property relationships, production of materials with precise structural control and advanced properties, and applications of bioinspired polymers for various fields including tissue engineering, drug delivery systems, or wound dressings [1,2,3,4].

The main problems of the resulting materials made from single polymers are insufficient mechanical properties and too rapid biodegradability. Therefore, mixtures of polymers and the use of a cell-free tissue matrix started to be used. Silk fibroin [5,6,7], collagen [8,9], hyaluronate [10,11], or gelatin [12] were used for this purpose. The materials obtained should be biodegradable and the biodegradation products must be non-toxic and removed from the body without any effect on other tissues. Furthermore, the materials should support cell adhesion, migration, and proliferation through appropriate porosity, pore size, and their appropriate combination. The physicochemical and mechanical properties should be as similar as possible to those of the tissue to be replaced and should be strong enough to allow its implantation during surgery [13,14]. These materials can take the form of thin films, hydrogels, membranes, 3D structures, fibers, and nanofibers.

Herein, we reviewed various examples of chitosan-based biomaterials, mixed with other polymers and cross-linked with chemical agents, in biomedical applications based on previous research.

## 2. Chitosan and Its Derivatives

**Figure 1:**
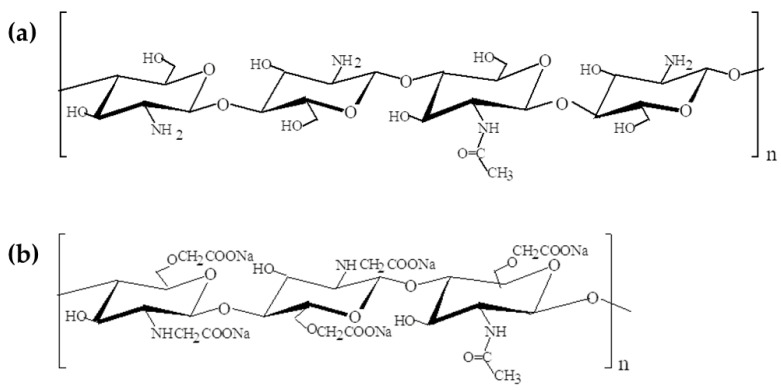
Structures of chitosan (**a**) and carboxymethyl chitosan (**b**).

Chitosan (CS) (poly(β-(1,4)-2-amino-2-deoxy-D-glucopyranose) is a natural polymer obtained by partial deacetylation of chitin in an alkaline medium (Figure 1). Chitin was produced from the exoskeletons of crustaceans. Chitosan also occurs naturally in the cell walls of some fungi. Chitosan is a polymer with a degree of deacetylation of at least 60%. The polymer’s molecular weight and the degree of deacetylation determine its properties such as biodegradability, biocompatibility, viscosity, hydrophilicity, and antibacterial or antifungal properties. The major disadvantage of chitosan is its lack of solubility in water [15,16,17,18,19,20,21].

**Figure 2:**
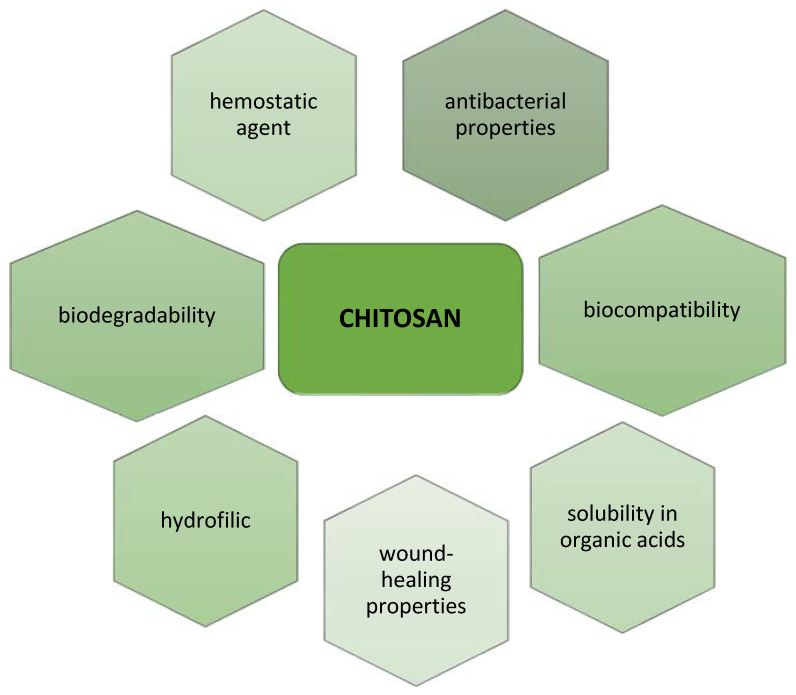
Chitosan properties.

The most important properties are shown in Figure 2. Chitosan is the only naturally occurring polysaccharide classified as a cationic polyelectrolyte, which allows it to interact with different types of molecules. The polymer’s positive charge is responsible for its antibacterial properties, attaching to the negatively charged cell membrane of various microorganisms [14,21].

Carboxymethyl chitosan (CMC) is a chitosan derivative in which the carboxymethyl group is attached to either an amino group or a hydroxyl group (Figure 1).

This chitosan derivative is water soluble and this is one of the main reasons for the increased interest in this polymer by researchers. It can be obtained in many types: N-carboxymethyl chitosan N,N-carboxymethyl chitosan, N,O-carboxymethyl chitosan, and O-carboxymethyl chitosan. During the substitution reaction, the listed types of derivatives or their mixtures can be obtained [22,23]. CMC is characterized by high viscosity, biocompatibility, and biodegradability, and is non-toxic. It also has antimicrobial activity, with O-carboxymethyl chitosan showing greater activity due to the more abundant presence of amino groups. Carboxymethyl chitosan shows improved physicochemical and biological properties relative to chitosan. The properties of CMC are influenced by the average molecular weight, degree of deacetylation, and degree of substitution. In addition, CMC has antioxidant activity, antibacterial or antifungal properties, and the ability to chelate metals [22,23,24,25,26]

Chitosan acetate is obtained by reaction with acetic acid in an aqueous–ethanol environment. It is water soluble and its solution is more stable than chitosan dissolved in acetic acid, while retaining the physicochemical and biological properties of chitosan. It exhibits stronger antimicrobial activity against Gram-positive bacteria than against Gram-negative bacteria. It is used as a dressing material (Chitopack C^®^) and a hemostat (Hemcon Bandage^®^) approved by the FDA [27].

There are other chitosan derivatives such as sulfopropylchitosan, O-quaternary ammonium salt of chitosan, N-succinylchitosan, and others [28,29].

## 3. Chitosan and Its Derivatives in Medicine

Due to its properties, chitosan and its derivatives can be used in the production of dressing materials, in the manufacture of drugs as a controlled-release active substance carrier, or in tissue engineering involving soft tissues, nerves, cartilage, bones, or arteries. Studies on the use of chitosan are summarized in Table 1 and studies on its derivatives are in Table 2.

The team of Fangsong Zhang et al. [30] used two chemical agents, glutaraldehyde, genipin, and a physical agent, ultraviolet light, to crosslink nerve extracellular matrix/chitosan scaffolds. Scaffolds cross-linked with genipin were characterized by higher porosity and regular structure in contrast to scaffolds cross-linked with glutaraldehyde and UV. The degree of crosslinking for genipin-crosslinked and glutaraldehyde-crosslinked scaffolds were similar to each other. Genipin-crosslinked scaffolds had the lowest degree of cytotoxicity and the highest histocompatibility, with good mechanical properties.

Another team, Jie Xu et al. [31], prepared a scaffold based on decellularized extracellular matrix, gelatin, and chitosan cross-linked EDC/NHS. The resulting scaffolds were characterized by a high modulus of elasticity and biodegradability. The obtained scaffolds are not cytotoxic and provided a good substrate for cell proliferation. The scaffolds were also characterized by antibacterial properties (*E. Coli*, *S. Aureus*). The scaffolds obtained could be used in skin tissue engineering.

A scaffold for use in muscle tissue engineering is a project by the team of Weiguang Zhao et al. [32]. They used genipin as a crosslinking agent and electrospun cellulose acetate nanofibers that were incorporated into a chitosan/fibroin silk cryogel scaffold. The resulting scaffolds were characterized by larger pores and roughness than the cryogel scaffold itself. They are also a good substrate for smooth muscle cell proliferation, which showed a higher potential for the expression of genes related to muscle contraction. They also exhibit good mechanical properties.

Scaffold for use in cartilage tissue engineering is a study by Christian E. G. Garcia et al. [33]. The properties of chitosan in two forms were compared: thin film and electrospinning scaffold chitosan/poly (ethylene oxide) (PEO). PEO of two different molecular weights was used and different weight ratios of Cs/PEO were applied. Some of the materials obtained were neutralized in order to compare the effect of neutralization on the properties of the scaffolds. The scaffolds after neutralization were characterized by better adhesion of chondrocyte cells and better proliferation; the worst properties were characterized by the chitosan film.

Nihui Zhang and her team [65] used genipin solutions with different concentrations (2.5%, 5%, 10%, and 15%) to produce carboxymethyl chitosan hydrogels. Analysis of HSFs cell proliferation and adhesion showed that the best cell adhesion and proliferation were obtained for the hydrogel with the highest amount of genipin, and the worst for the hydrogel with the lowest proportion of genipin. The best properties for promoting wound healing and reducing the appearance of scars in vivo tests were obtained for the hydrogel carboxymethyl chitosan/genipin 5% (*v/v*). This was confirmed by an in vivo test using female rats. With the additional contribution of aloe vera gel, wound healing results improved even further. In conclusion, genipin-crosslinked chitosan hydrogels are promising candidates for use as a dressing to accelerate wound healing.

Yalei Liu et al. [66] used polyvinyl alcohol, carboxymethyl chitosan, silver nanoparticles, and borax as a crosslinking agent to produce a hydrogel. The resulting hydrogel, due to its dual crosslinking (hydrogen bonds and borate ester bonds), has self-healing properties and is characterized by good mechanical properties. It also exhibits antibacterial properties, as confirmed by a test with *E.coli* and *S. aureus* bacteria. A cytotoxicity test was also performed using L929 cells, which showed that the resulting scaffolds were non-toxic.

Guozhu Chang et al. [67] produced a carboxymethyl chitosan/carboxymethyl cellulose hydrogel using heparin and glutaraldehyde as a crosslinking agent. This allowed the fabrication of a self-healing hydrogel. It is biocompatible with cells and its ability to release drugs has also been studied. An in vivo study was also performed on rats with diabetes, where its effect on accelerating open wound healing was confirmed. It can be concluded that the resulting hydrogel has the potential to be used as a material to accelerate diabetic wound healing.

To form antimicrobial scaffolds, A. Mishra’s team [68] used carboxymethyl chitosan, zinc, and genipin. Carboxymethyl chitosan/genipin/Zn scaffolds were obtained. Wet compression analysis showed that the carboxymethyl chitosan/genipin/Zn scaffold was more robust than the non-cross-linked scaffold. Degradation testing was carried out under enzymatic and non-enzymatic conditions. The resulting scaffold also showed good stability. An adhesion and proliferation test was performed using dental pulp stem cells; in addition, a biocompatibility test against red blood cells was performed, which confirmed its good biological properties. An antibacterial test was also performed *(Pseudomonas aeruginosa* ATCC 25619, *S. aureus* ATCC 9144, *S. aureus* ATCC 25923, and *Staphylococcus epidermidis* ATCC 155). No biofilm formed on the surface of the scaffold carboxymethyl chitosan/genipin/Zn. In conclusion, scaffold carboxymethyl chitosan/genipin/Zn can find application in dental tissue engineering due to its antibacterial properties.

**Figure 3:**
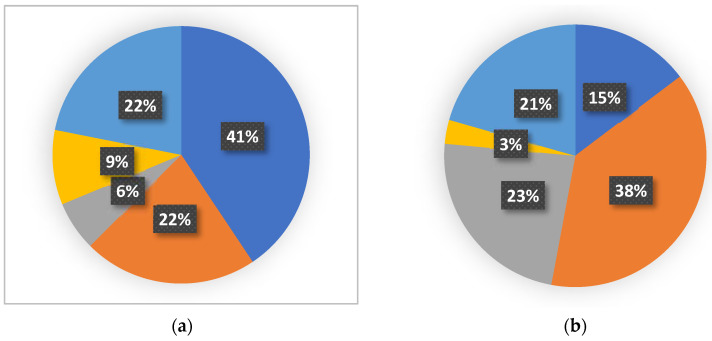
Applications of chitosan (**a**) and its derivatives (**b**) in tissue engineering. Skin tissue engineering (dark blue), bone tissue engineering (orange), cartilage tissue engineering (grey), dental tissue engineering (yellow), and other tissue engineering and unclassified tissue engineering (light blue).

Summarizing the data overview on the use of chitosan and its derivatives in tissue engineering (Figure 3a,b), it can be written that the most research on chitosan-based materials concerned bone tissue engineering and the least concerned dental tissue engineering. For materials based on chitosan derivatives, the greatest interest in use was in skin tissue engineering and the least in tissue engineering applications.

## 4. Conclusions

The use of chitosan and its derivatives in medicine offers a huge opportunity in the further development of regenerative medicine. The use of different forms of polymers such as films, hydrogel scaffolds, or the use of strongly developing ways of producing materials such as electrospinning and 3D printing open another door to the medicine of the future. Owing to continuous development, we are able to produce biomaterials that mimic the structure, morphology, and function of various organs such as blood vessels, nerves, soft tissues, or bones. Further research using other solvents, new mixtures, or using a different cross-linking agent may bring us even closer to a perfectly mimicking tissue biomaterial. A constant challenge is to produce in the spirit of green production and ecology in a closed loop using natural polymers where their extraction will not adversely affect the environment.

## Figures and Tables

**Table 1:** Application of chitosan-based materials in tissue engineering.

Composition	Method	Application	In Vivo/In Vitro	Advantages	Ref.
Chitosan, genipin	Crosslinking, freeze-drying	Spinal cord tissue engineering	In vivo (rats)	Low cytotoxicity, high histocompatibility, good mechanical properties	[30]
Decellularized extracellular matrix/gelatin/and chitosan, EDC/NHS	Crosslinking, freeze-drying	Skin tissue engineering	In vitro (L929 fibroblasts)	The high modulus of elasticity, biodegradability, non-cytotoxic	[31]
Cellulose acetate nanofibers/chitosan/fibroin silk cryogel scaffold, genipin	Electrospinning, crosslinking, freeze-drying	Smooth muscle tissue engineering	In vitro (smooth muscle cell)	Good mechanical properties, good proliferation	[32]
Chitosan/poly (ethylene oxide)	Electrospinning scaffold	Cartilage tissue engineering	In vitro (chondrocyte cells)	Good cell adhesion and proliferation	[33]
Hyaluronic acid/chitosan coacervate-based scaffolds	Centrifuge, incubation	Cartilage tissue engineering	In vitro	Good proliferation and cell viability	[34]
PCL/chitosan-PEO with *A. euchroma* extract	Two-nozzle electrospinning	Skin tissue engineering	In vitro (HDF cells)	Good proliferation and cell viability	[35]
Hydrogels of chitosan/oxidized-modified quince seed gum/curcumin-loaded	Encapsulation	Tissue engineering	In vitro (NIH3T3 fibroblast cells)	Improved thermal stability, swelling ratio, and degradation rate of hydrogels, non-cytotoxicity, good proliferation	[36]
Chitosan scaffolds, sodium hydroxide-crosslinking agent	3D print	Cartilage tissue engineering	In vitro (ATDC5 cells)	Higher elastic modulus, good biocompatibility	[37]
Gelatin/chitosan/polyvinyl alcohol/nano-hydroxyapatite	Freeze-drying	Bone tissue engineering	In vitro (BMSCs cells)	Improved surface bioactivity and biomimetic structure, high osteogenic differentiation ability	[38]
Polycaprolactone–polyurethane/chitosan	Freeze-drying, drying in oven	Bone tissue engineering	In vitro (hBMSCs)	Non-cytotoxicity, good mechanical properties, good promotion of the formation of calcium levels, good gene expression	[39]
Chitosan–hydroxyapatite–carbon	Drying in oven	Bone tissue engineering	In vitro (human osteoblasts)	Good biocompatibility with human osteoblasts, good mechanical properties	[40]
Polycaprolactone/chitosan-g-polycaprolactone/hydroxyapatite	Electrospinning, drying in oven	Bone tissue engineering	In vitro (NIH3T3 fibroblast cells)	High cell viability and proliferation, good mechanical properties	[41]
Chitosan–vanillin–BG (CVB)	Freeze-drying	Bone tissue engineering	In vivo (female mice	Good biocompatibility, bioactivity, strong antibacterial ability, good promotion of	[42]
				osteoblastic differentiation, ectopic bone formation in vivo	
Chitosan-pyrolyzed cork	Freeze-drying	Electrically active biological tissue engineering	In vitro (SH-SY5Y neuroblastoma cell)	Good biocompatibility, high mechanical strength	[43]
Polycaprolactone (PCL)–chitosan/carboxyl carbon	Electrospinning	Cartilage tissue engineering	In vitro (chondrocytes cells)	High porosity, good mechanical properties, good biocompatibility	[44]
Decellularized Alstroemeria flower stem/chitosan	Freeze-drying	Tissue engineering	In vitro (MC3T3 cells)	Good cell attachment, proliferation and migration, good mechanical properties	[45]
Chitosan/hydroxypropyl methyl cellulose/hydroxyapatite/ lemon grass oil	Freeze gelation method	Bone tissue engineering	In vitro (MC3T3 cells)	Antimicrobial activity (*S. aureus*), non-toxic	[46]
Chitosan/βGP/NaHCO3/ HAp/PECs/gelatin	Gelation in a water batch	Bone tissue engineering	In vitro (MG63 cells)	Good cellular proliferation, osteogenic differentiation	[47]
Chitosan–tripolyphosphate	Exploiting dialysis technique, freeze-drying	Tissue engineering	In vitro (NIH3T3 fibroblast cells)	Good biocompatibility, good mechanical properties	[48]
Chitosan scaffolds with controllable microchannel	Combining a 3D printing microfiber template-leaching method and a freeze-drying method	Tissue engineering	In vitro (NIH3T3 fibroblast cells), in vivo (rats)	Good cell proliferation and distribution, improved cell, tissue growth and vascular formation	[49]
Chitosan/loofah/Poly(3-hydroxybutyric acid-co-3-hydroxyvaleric acid)	Electrospinning, freeze-drying	Tissue engineering	In vitro (human mesenchymal stem cells)	Good cell proliferation and migration, good mechanical and	[50]
				viscoelastic properties, differentiation into adipogenic, osteogenic, and chondrogenic tissues	
Xylan/chitosan/nano-HAp/graphene oxide/reduced graphene oxide	Freeze-drying	Bone tissue engineering	In vitro (MG-63 cell)	Improved mineralization tendency, osteogenic differentiation capability	[51]
Hybrid bionanocomposite of chitosan/poly(vinyl alcohol)/nanobioactive glass/nanocellulose	Drying in oven	Bone tissue engineering	In vitro (red blood cells)	Good porosity, better antibacterial effect (*E. coli, S. aureus*), improved hemocompatibility	[52]
Bacterial cellulose/chitosan/alginate/gelatin	Stirring with heat	Cartilage tissue engineering	In vitro (human mesenchymal stem cells)	Good compressive strength, stability, biocompatibility, good cell proliferation	[53]
Chitosan/poly(vinyl alcohol)/nano bioactive glass/nano zinc oxide	Drying in oven	Bone tissue engineering	In vitro (red blood cells)	Better tensile strength, good hemocompatibility, antimicrobial activity (*Enterococcus faecalis, Salmonella typhi*)	[54]
Calcium silicate-coated porous chitosan	Freeze-drying	Dental tissue engineering	In vitro (human dental pulp cells)	Good cell proliferation and mineralization	[55]
Graphene-oxide-containing chitosan	Freeze-drying	Cartilage tissue engineering	In vitro (chondro-cytes cells)	Improved physical and mechanical properties, good proliferation	[56]
Injectable chitosan/beta glycerophosphate/pyrrole oligomers	Stirring	Cartilage tissue engineering	In vitro (fibroblastoid cell CHO-K1)	Good biodegradability, biocompatibility, electro-activity, swelling ratio, and pore size values	[57]
Silk fibroin–chitosan	Freeze-drying	Cartilage tissue engineering	In vitro (human mesenchymal stem cell)	Good porosity, good compressive strength, proliferation, cell viability	[58]
Chitosan/ modified montmorillonite/hydroxyapatite	Microwave irradiation, gas-foaming method, freeze-drying	Bone tissue engineering	In vitro (MG 63 osteoblast cell)	Non-cytotoxic, good biodegradation, swelling properties, and good mechanical properties	[59]
Chitosan-grafted-poly(methyl methacrylate)/hydroxyapatite scaffold	Freeze-drying	Bone tissue engineering	In vitro (UMR-106 osteoblast-like cells)	Good viability, proliferation, and cells attachment, good mechanical properties, good drug delivery	[60]
Poly-L-lactic acid/chitosan/collagen	Electrospinning	Vascular tissue engineering	In vitro (lymphocyte T cell)	Good cell viability and hemolysis, good mechanical properties, and bust pressure	[61]
Gelatin/chitosan	Electrospinning	Skin tissue engineering	In vitro (human dermal fibroblast cells)	Very good porosity, good mechanical properties, non-cytotoxic, spindle-like shape cells	[62]
l-chitosan/maleic terminated polyethylene glycol	Freeze-drying	Skin tissue engineering	In vitro (HFFF2 cells), in vivo (rats)	Porous structure, high swelling ratio, biocompatibility, fully closed wound with improved vascularization	[63]
Chitosan–vitamin C–lactic acid	Freeze-drying	Skin tissue engineering	In vitro (NIH3T3 fibroblast cells)	Good cell attachment, proliferation and spreading	[64]

**Table 2:** Applications of chitosan-derivative-based materials in tissue engineering.

Composition	Method	Application	In Vivo/In Vitro	Advantages	Ref.
Carboxymethyl chitosan/genipin	Stirring	Skin tissue engineering	In vitro (HSFs cells) in vivo (rats)	Good cell attachment and proliferation, good wound healing promotion	[65]
Polyvinyl alcohol, carboxymethyl chitosan with silver nanoparticles and borax	Stirring	Skin tissue engineering	In vitro (L929 cells)	Antibacterial properties, good mechanical properties, non-cytotoxic	[66]
Carboxymethyl chitosan/carboxymethyl cellulose hydrogel with heparin and glutaraldehyde	Stirring	Skin tissue engineering	In vivo (rats with diabetes)	Accelerated open wound healing	[67]
Carboxymethyl chitosan/genipin/Zn scaffolds	Freeze-drying	Dental tissue engineering	In vitro (dental pulp stem cells)	Antibacterial properties, good cell proliferation	[68]
Thiolated chitosan and silk fibroin	Incubating at 37 °C	Cartilage tissue engineering	In vitro (chondrocytes cells)	Good mechanical properties, high porosity, good cell proliferation	[69]
Lactoferrin-loaded carboxymethyl cellulose glycol chitosan	Stirring, 3D printing	Tissue engineering applications	In vitro (mouse osteoblastic cells)	Good biocompatibility, good physician properties	[70]
Silk fibroin/carboxymethyl chitosan hydrogel crosslinking by horseradish peroxidase	Stirring	Cartilage tissue engineering	In vitro (chondrocytes cells)	Good biocompatibility, biodegradability, good mechanical and rheological properties	[71]
Carboxymethyl chitosan/oxidized pullulan with methotrexate-loaded mesoporous silica	Stirring	Drug delivery	In vitro (human hepatoma SMMC-7721 and hepatic LO2 cells)	Good biocompatibility, non-cytotoxic, good drug release	[72]
Polymerized CMC-modified adhesive	Mixing the powder with the adhesive	Dental tissue engineering	Antibacterial test	Good antibacterial properties (*S. mutans*)	[73]
Oxidized microcrystalline cellulose/ carboxymethyl chitosan	Stirring	Skin tissue engineering	In vitro blood compatibility test	Good mechanical, self-healing characteristic, good coagulation	[74]
Silk fibroin/carboxymethyl chitosan/strontium substituted hydroxyapatite/cellulose	Freeze-drying	Bone tissue engineering	In vitro (BMSCs cells)	Non-toxic, good hemocompatibility, good gene expression (osteogenic gene markers), high porosity	[75]
Carboxymethyl chitosan-modified glass ionomer cement	Mixing	Dental tissue engineering	In vitro (NIH 3 T3 fibroblast cells)	Good biocompatibility, good attachment, and cell proliferation, better mechanical properties	[76]
Poly(3,4-ethylenedioxythiophene)/ carboxymethyl chitosan	Vibration	Neural tissue engineering		Good biodegradation and electroconductivity, good compressive modulus, better cell adhesion, viability and proliferation	[77]
Benzaldehyde-terminated 4-arm PEG/carboxymethyl chitosan/basic fibroblast growth factor	Stirring	Skin tissue engineering	In vitro (blood cells)	Excellent biocompatibility, fast hemostasis capacity, strong wet-tissue adhesion, self-mending, and antibacterial property	[78]
Polycaprolactone /carboxymethyl chitosan/sodium alginate micron-fibrous	Emulsion electrospinning	Periosteal tissue engineering	In vitro (osteoblasts cells)	Excellent tensile strength, no significant cytotoxicity, good cell adhesion	[79]
Carboxymethyl chitosan/sodium alginate hydrogels with polydopamine coatings	Immersion	Skin tissue engineering	In vitro (human umbilical vein endothelial cells), in vivo (rats with MRSA)	Antibacterial, anti-inflammatory properties, good antibacterial properties (*Methicillin-resistant Staphylococcus aureus*), fast wound healing	[80]
Chitosan/carboxymethyl cellulose with silver nanoparticles	Stirring	Skin tissue engineering	In vitro (human skin fibroblasts)	Good mechanical properties, good antibacterial properties (*E.coli*), non-cytotoxic	[81]
Gelatin/carboxymethyl chitosan/nano-hydroxyapatite	Freeze-drying	Bone tissue engineering	In vitro (human Wharton’s jelly MSC microtissue)	High porosity, slow enzymatic degradation, good mechanical properties, good viability, the proliferation of human Wharton’s jelly MSC microtissue	[82]
N,O-carboxymethyl chitosan/fucoidan	Freeze-drying	Bone tissue engineering	In vitro (L929 cells)	Good mineralization, good physical properties, good cell proliferation and mineralization	[83]
Diselenide-crosslinked carboxymethyl chitosan nanoparticles with doxorubicin	Stirring, dialysis	Drug delivery	In vitro (tumor cells)	High drug encapsulation efficiency, high drug accumulation, and cytotoxicity in tumor cells	[84]
Thiolated carboxymethyl chitosan-based 3D scaffolds	Freeze-drying	Theragnostic of tissue regeneration	In vitro (human dermo fibroblast cells)	High porosity, good mechanical properties, non-cytotoxic	[85]
Quaternized chitosan/hydroxyapatite curcumin-loaded	Stirring	Bone tissue engineering	In vitro (MG-63 cells)	Good mechanical strength, drug release, good biocompatibility and cell proliferation	[86]
Carboxymethyl chitosan/cellulose nanofiber	Freeze-drying, drying in the oven	Skin tissue engineering	In vivo (rats)	Good blood absorption, and excellent coagulation ability	[87]
Carboxymethyl chitosan–plantamajoside	Stirring	Skin tissue engineering	In vitro (L929 cells), in vivo (rats with burn wounds)	Good porosity, good cell viability, proliferation, significantly improved wound healing, granulation tissue proliferation	[88]
Polycaprolactone/galactosylated chitosan	Freeze-drying, electrospinning	Liver tissue engineering	In vitro (HepG2 cells)	Non-cytotoxic, good cell growth, and proliferation	[89]
Cotton fabric/carboxymethyl chitosan/silver nitrate	Pad–dry–cure method, drying in oven	Skin tissue engineering	In vivo (rats with wounds)	Good wound healing properties, antibacterial properties (*E. coli, S. aureus*)	[90]
Chitosan–gelatin–hyaluronic acid	Freeze-drying	Skin tissue engineering	In vitro (fibroblast and keratinocytes cells)	Good mechanical properties, flexible scaffold/cells, artificial skin, good cell proliferation in co-cultures	[91]
Mannose-anchored quaternized chitosan/thiolated carboxymethyl chitosan	Freeze-drying	Drug delivery	In vitro (293T cells)	Non-cytotoxic, high hydrophilicity, good drug release and stability	[92]
Chitosan, carboxymethyl cellulose and silver-nanoparticle-modified cellulose nanowhiskers	Freeze-drying	Bone tissue engineering	In vitro (MG63 cells)	Good mechanical properties, high porosity, excellent antimicrobial activity (*E. coli*), good biomineralization	[93]
N, O-carboxymethyl chitosan/oxidized cellulose containing ε-poly-L-lysine	Freeze-drying	Skin tissue engineering	In vitro (NIH 3T3 cells), in vivo (rabbit)	Good antibacterial properties (*E. coli, S. aureus*), excellent biological security and compatibility in vitro and in vivo	[94]
O-carboxymethyl chitosan/sodium alginate with insulin	Stirring	Drug delivery	In vitro (L929 mouse fibroblast cells), in vivo (rats)	High drug loading capacity and high effectively released drugs as oral drugs, lower glucose level compared with insulin injections	[95]
Polycaprolactone/carboxymethyl chitosan	Electrospinning	Bone tissue engineering	In vitro (human osteoblast cells MG63)	Good biocompatibility, good cell proliferation	[96]
O-carboxymethyl chitosan nonwoven fabrics	Chitosan needle-punched nonwoven reaction with chloroacetic acid	Skin tissue engineering	In vitro (L929 mouse fibroblast cells), in vivo (rats with a partial-thickness burn)	Good mechanical properties, good cell migration, and proliferation, good healing rate, good angiogenesis	[97]
Recombinant human collagen/carboxylated chitosan	Stirring	Soft tissue engineering	In vitro (NIH 3T3 cells), in vivo (rats with open wounds)	Good biocompatibility, non-cytotoxic, acceleration of the cell infiltration and wound closure	[98]
Nano-hydroxyapatite/chitosan/polyethylene glycol	Stirring, filtration, drying in the oven	Bone tissue engineering	In vitro (murine fibroblast L929 cells)	Good thermal stability and swelling ratio, non-cytotoxic	[99]
Norcantharidin-conjugated carboxymethyl chitosan	Vacuum-dried	Drug delivery	In vitro (BEL-7402 cells), in vivo (mice with H22 cells, tumor cells)	Inhibitory effects on the proliferation and migration of cells, changes in cell structure, reduction in the distribution of norcantharidin in heart and kidney tissues, diminished systemic toxicity	[100]
Poly (vinyl alcohol) and fungal mushroom-derived carboxymethyl chitosan	Solution casting technique	Skin tissue engineering	In vitro (skin fibroblasts and keratinocytes)	Good antibacterial properties (*E. coli, S. aureus*), good biocompatibility, good hemolysis	[101]
Carboxymethyl chitosan/oxidized dextran/sodium alginate	Mixing with a double-barreled syringe	Skin tissue engineering	In vitro (L929 cells), in vivo (rat liver injury model and mouse tail amputation model)	Red blood cells could adhere to the surface of hydrogel, good hemostasis, good antibacterial properties (*S. aureus*)	[102]
N,O-carboxymethyl chitosan	Stirring	Drug delivery	In vivo (rabbit)	Good drug delivery, non-cytotoxic to the cornea, good degradability	[103]

## Data Availability

Not applicable.

## References

[1] Zhao X., He X., Hou A., Cheng C., Wang X., Yue Y., Wu Z., Wu H., Liu B., Li H. (2022). Growth of Cu2O Nanoparticles on Two-Dimensional Zr–Ferrocene–Metal–Organic Framework Nanosheets for Photothermally Enhanced Chemodynamic Antibacterial Therapy. Inorg. Chem..

[2] Zhou Z., Wang Y., Peng F., Meng F., Zha J., Ma L., Du Y., Peng N., Ma L., Zhang Q. (2022). Intercalation-Activated Layered MoO3 Nanobelts as Biodegradable Nanozymes for Tumor-Specific Photo-Enhanced Catalytic Therapy. Angew. Chem. Int. Ed..

[3] Qin L., Liang F., Li Y., Wu J., Guan S., Wu M., Xie S., Luo M., Ma D. (2022). A 2D Porous Zinc-Organic Framework Platform for Loading of 5-Fluorouracil. Inorganics.

[4] Hu T., Gu Z., Williams G.R., Strimaite M., Zha J., Zhou Z., Zhang X., Tan C., Liang R. (2022). Layered Double Hydroxide-Based Nanomaterials for Biomedical Applications. Chem. Soc. Rev..

[5] Tu D.D., Chung Y.G., Gil E.S., Seth A., Franck D., Cristofaro V., Sullivan M.P., Di Vizio D., Gomez P., Adam R.M. (2013). Bladder Tissue Regeneration Using Acellular Bi-Layer Silk Scaffolds in a Large Animal Model of Augmentation Cystoplasty. Biomaterials.

[6] Xiao S., Wang P., Zhao J., Ling Z., An Z., Fu Z., Fu W., Zhang X. (2021). Bi-Layer Silk Fibroin Skeleton and Bladder Acellular Matrix Hydrogel Encapsulating Adipose-Derived Stem Cells for Bladder Reconstruction. Biomater. Sci..

[7] Cao N., Song L., Liu W., Fan S., Jiang D., Mu J., Gu B., Xu Y., Zhang Y., Huang J. (2018). Prevascularized Bladder Acellular Matrix Hydrogel/Silk Fibroin Composite Scaffolds Promote the Regeneration of Urethra in a Rabbit Model. Biomed. Mater..

[8] Gasanz C., Raventós C., Temprana-Salvador J., Esteves M., Fonseca C., de Torres I., Morote J. (2018). Use of an Acellular Collagen–Elastin Matrix to Support Bladder Regeneration in a Porcine Model of Peritoneocystoplasty. Cent. Eur. J. Urol..

[9] Shi C., Chen W., Chen B., Shan T., Jia W., Hou X., Li L., Ye G., Dai J. (2017). Bladder Regeneration in a Canine Model Using a Bladder Acellular Matrix Loaded with a Collagen-Binding BFGF. Biomater. Sci..

[10] Jalali S., Fereidoni M., Shahri N.M., Lari R. (2019). Effect of Swim Bladder Matrix Treated with Hyaluronic Acid on Wound Healing: An Animal Model Evaluation. J. Wound Care.

[11] Su Z., Ma H., Wu Z., Zeng H., Li Z., Wang Y., Liu G., Xu B., Lin Y., Zhang P. (2014). Enhancement of Skin Wound Healing with Decellularized Scaffolds Loaded with Hyaluronic Acid and Epidermal Growth Factor. Mater. Sci. Eng. C.

[12] Mathapati S., Bishi D.K., Venugopal J.R., Cherian K.M., Guhathakurta S., Ramakrishna S., Verma R.S. (2014). Nanofibers Coated on Acellular Tissue-Engineered Bovine Pericardium Supports Differentiation of Mesenchymal Stem Cells into Endothelial Cells for Tissue Engineering. Nanomedicine.

[13] Dolcimascolo A., Calabrese G., Conoci S., Parenti R., Barbeck M., Jung O., Smeets R., Koržinskas T. (2019). Innovative Biomaterials for Tissue Engineering. Biomaterial-Supported Tissue Reconstruction or Regeneration.

[14] Rodríguez-Vázquez M., Vega-Ruiz B., Ramos-Zúñiga R., Saldaña-Koppel D.A., Quiñones-Olvera L.F. (2015). Chitosan and Its Potential Use as a Scaffold for Tissue Engineering in Regenerative Medicine. BioMed Res. Int..

[15] Huang Y., Onyeri S., Siewe M., Moshfeghian A., Madihally S.V. (2005). In Vitro Characterization of Chitosan–Gelatin Scaffolds for Tissue Engineering. Biomaterials.

[16] Sencadas V., Correia D.M., Ribeiro C., Moreira S., Botelho G., Gómez Ribelles J.L., Lanceros-Mendez S. (2012). Physical-Chemical Properties of Cross-Linked Chitosan Electrospun Fiber Mats. Polym. Test..

[17] Muxika A., Etxabide A., Uranga J., Guerrero P., de la Caba K. (2017). Chitosan as a Bioactive Polymer: Processing, Properties and Applications. Int. J. Biol. Macromol..

[18] Aguilar A., Zein N., Harmouch E., Hafdi B., Bornert F., Offner D., Clauss F., Fioretti F., Huck O., Benkirane-Jessel N. (2019). Application of Chitosan in Bone and Dental Engineering. Molecules.

[19] Baranwal A., Kumar A., Priyadharshini A., Oggu G.S., Bhatnagar I., Srivastava A., Chandra P. (2018). Chitosan: An Undisputed Bio-Fabrication Material for Tissue Engineering and Bio-Sensing Applications. Int. J. Biol. Macromol..

[20] El Knidri H., Belaabed R., Addaou A., Laajeb A., Lahsini A. (2018). Extraction, Chemical Modification and Characterization of Chitin and Chitosan. Int. J. Biol. Macromol..

[21] Yang J., Tian F., Wang Z., Wang Q., Zeng Y.-J., Chen S.-Q. (2008). Effect of Chitosan Molecular Weight and Deacetylation Degree on Hemostasis. J. Biomed. Mater. Res..

[22] Jimtaisong A., Saewan N. (2014). Utilization of Carboxymethyl Chitosan in Cosmetics. Int. J. Cosmet. Sci..

[23] Muzzarelli R.A.A. (1988). Carboxymethylated Chitins and Chitosans. Carbohydr. Polym..

[24] Mourya V.K., Inamdara N., Ashutosh Tiwari N. (2010). Carboxymethyl Chitosan and Its Applications. Adv. Mater. Lett..

[25] Sun T., Yao Q., Zhou D., Mao F. (2008). Antioxidant Activity of N-Carboxymethyl Chitosan Oligosaccharides. Bioorg. Med. Chem. Lett..

[26] Shariatinia Z. (2018). Carboxymethyl Chitosan: Properties and Biomedical Applications. Int. J. Biol. Macromol..

[27] Li Y., Chen X.G., Liu N., Liu C.S., Liu C.G., Meng X.H., Yu L.J., Kenendy J.F. (2007). Physicochemical Characterization and Antibacterial Property of Chitosan Acetates. Carbohydr. Polym..

[28] Kahya N. (2019). Water Soluble Chitosan Derivatives and Their Biological Activities: A Review. Polym. Sci..

[29] Kato Y. (2004). N-Succinyl-Chitosan as a Drug Carrier: Water-Insoluble and Water-Soluble Conjugates. Biomaterials.

[30] Zhang F., Zhang N., Xu Q., Zhang L., Zhang C., Liu H., Yu Z., Zhou S., Feng G., Huang F. (2021). Decellularized Nerve Extracellular Matrix/Chitosan Crosslinked by Genipin to Prepare a Moldable Nerve Repair Material. Cell Tissue Bank.

[31] Xu J., Fang H., Zheng S., Li L., Jiao Z., Wang H., Nie Y., Liu T., Song K. (2021). A Biological Functional Hybrid Scaffold Based on Decellularized Extracellular Matrix/Gelatin/Chitosan with High Biocompatibility and Antibacterial Activity for Skin Tissue Engineering. Int. J. Biol. Macromol..

[32] Zhao W., Cao S., Cai H., Wu Y., Pan Q., Lin H., Fang J., He Y., Deng H., Liu Z. (2022). Chitosan/Silk Fibroin Biomimic Scaffolds Reinforced by Cellulose Acetate Nanofibers for Smooth Muscle Tissue Engineering. Carbohydr. Polym..

[33] Garcia C.E.G., Lardy B., Bossard F., Soltero F.A. (2021). Chitosan based biomaterials for cartilage tissue engineering: Chondrocyte adhesion and proliferation. Food Hydrocolloids for Health.

[34] Karabıyık Acar Ö., Bedir S., Kayitmazer A.B., Kose G.T. (2021). Chondro-Inductive Hyaluronic Acid/Chitosan Coacervate-Based Scaffolds for Cartilage Tissue Engineering. Int. J. Biol. Macromol..

[35] Asghari F., Rabiei Faradonbeh D., Malekshahi Z.V., Nekounam H., Ghaemi B., Yousefpoor Y., Ghanbari H., Faridi-Majidi R. (2022). Hybrid PCL/Chitosan-PEO Nanofibrous Scaffolds Incorporated with A. Euchroma Extract for Skin Tissue Engineering Application. Carbohydr. Polym..

[36] Yavari Maroufi L., Ghorbani M. (2021). Injectable Chitosan-Quince Seed Gum Hydrogels Encapsulated with Curcumin Loaded-Halloysite Nanotubes Designed for Tissue Engineering Application. Int. J. Biol. Macromol..

[37] Sadeghianmaryan A., Naghieh S., Alizadeh Sardroud H., Yazdanpanah Z., Afzal Soltani Y., Sernaglia J., Chen X. (2020). Extrusion-Based Printing of Chitosan Scaffolds and Their in Vitro Characterization for Cartilage Tissue Engineering. Int. J. Biol. Macromol..

[38] Ma P., Wu W., Wei Y., Ren L., Lin S., Wu J. (2021). Biomimetic Gelatin/Chitosan/Polyvinyl Alcohol/Nano-Hydroxyapatite Scaffolds for Bone Tissue Engineering. Mater. Des..

[39] Amiryaghoubi N., Noroozi Pesyan N., Fathi M., Omidi Y. (2022). The Design of Polycaprolactone-Polyurethane/Chitosan Composite for Bone Tissue Engineering. Colloids Surf. A Physicochem. Eng. Asp..

[40] Sanchez A.G., Prokhorov E., Luna-Barcenas G., Hernández-Vargas J., Román-Doval R., Mendoza S., Rojas-Chávez H. (2021). Chitosan-Hydroxyapatite-MWCNTs Nanocomposite Patch for Bone Tissue Engineering Applications. Mater. Today Commun..

[41] Shirzaei Sani I., Rezaei M., Baradar Khoshfetrat A., Razzaghi D. (2021). Preparation and Characterization of Polycaprolactone/Chitosan-g-Polycaprolactone/Hydroxyapatite Electrospun Nanocomposite Scaffolds for Bone Tissue Engineering. Int. J. Biol. Macromol..

[42] Hu J., Wang Z., Miszuk J.M., Zhu M., Lansakara T.I., Tivanski A.V., Banas J.A., Sun H. (2021). Vanillin-Bioglass Cross-Linked 3D Porous Chitosan Scaffolds with Strong Osteopromotive and Antibacterial Abilities for Bone Tissue Engineering. Carbohydr. Polym..

[43] Scalera F., Monteduro A.G., Maruccio G., Blasi L., Gervaso F., Mazzotta E., Malitesta C., Piccirillo C. (2021). Sustainable Chitosan-Based Electrical Responsive Scaffolds for Tissue Engineering Applications. Sustain. Mater. Technol..

[44] Mirmusavi M.H., Ahmadian M., Karbasi S. (2022). Polycaprolactone-Chitosan/Multi-Walled Carbon Nanotube: A Highly Strengthened Electrospun Nanocomposite Scaffold for Cartilage Tissue Engineering. Int. J. Biol. Macromol..

[45] Esmaeili J., Jadbabaee S., Far F.M., Lukolayeh M.E., Kırboğa K.K., Rezaei F.S., Barati A. (2022). Decellularized Alstroemeria Flower Stem Modified with Chitosan for Tissue Engineering Purposes: A Cellulose/Chitosan Scaffold. Int. J. Biol. Macromol..

[46] Ali H.U., Iqbal D.N., Iqbal M., Ezzine S., Arshad A., Zeeshan R., Chaudhry A.A., Alshawwa S.Z., Nazir A., Khan A.F. (2022). HPMC Crosslinked Chitosan/Hydroxyapatite Scaffolds Containing Lemongrass Oil for Potential Bone Tissue Engineering Applications. Arab. J. Chem..

[47] Wasupalli G.K., Verma D. (2022). Thermosensitive Injectable Hydrogel Based on Chitosan-Polygalacturonic Acid Polyelectrolyte Complexes for Bone Tissue Engineering. Carbohydr. Polym..

[48] Sacco P., Borgogna M., Travan A., Marsich E., Paoletti S., Asaro F., Grassi M., Donati I. (2014). Polysaccharide-Based Networks from Homogeneous Chitosan-Tripolyphosphate Hydrogels: Synthesis and Characterization. Biomacromolecules.

[49] Jiang Z., Zhang K., Du L., Cheng Z., Zhang T., Ding J., Li W., Xu B., Zhu M. (2021). Construction of Chitosan Scaffolds with Controllable Microchannel for Tissue Engineering and Regenerative Medicine. Mater. Sci. Eng. C.

[50] Baysan G., Colpankan Gunes O., Akokay P., Husemoglu R.B., Ertugruloglu P., Ziylan Albayrak A., Cecen B., Havitcioglu H. (2022). Loofah-Chitosan and Poly (−3-Hydroxybutyrate-Co-3-Hydroxyvalerate) (PHBV) Based Hydrogel Scaffolds for Meniscus Tissue Engineering Applications. Int. J. Biol. Macromol..

[51] Ali A., Hasan A., Negi Y.S. (2022). Effect of Carbon Based Fillers on Xylan/Chitosan/Nano-HAp Composite Matrix for Bone Tissue Engineering Application. Int. J. Biol. Macromol..

[52] Narmatha C.P., Khaleel B.S., Sugantha K.V. (2022). Multifunctional Organic and Inorganic Hybrid Bionanocomposite of Chitosan/Poly(Vinyl Alcohol)/Nanobioactive Glass/Nanocellulose for Bone Tissue Engineering. J. Mech. Behav. Biomed. Mater..

[53] Phatchayawat P.P., Khamkeaw A., Yodmuang S., Phisalaphong M. (2022). 3D Bacterial Cellulose-Chitosan-Alginate-Gelatin Hydrogel Scaffold for Cartilage Tissue Engineering. Biochem. Eng. J..

[54] Christy P.N., Basha S.K., Kumari V.S. (2022). Nano Zinc Oxide and Nano Bioactive Glass Reinforced Chitosan/Poly(Vinyl Alcohol) Scaffolds for Bone Tissue Engineering Application. Mater. Today Commun..

[55] Leite M.L., Anselmi C., Soares I.P.M., Manso A.P., Hebling J., Carvalho R.M., de Souza Costa C.A. (2022). Calcium Silicate-Coated Porous Chitosan Scaffold as a Cell-Free Tissue Engineering System for Direct Pulp Capping. Dent. Mater..

[56] Shamekhi M.A., Mirzadeh H., Mahdavi H., Rabiee A., Mohebbi-Kalhori D., Baghaban Eslaminejad M. (2019). Graphene Oxide Containing Chitosan Scaffolds for Cartilage Tissue Engineering. Int. J. Biol. Macromol..

[57] Kashi M., Baghbani F., Moztarzadeh F., Mobasheri H., Kowsari E. (2018). Green Synthesis of Degradable Conductive Thermosensitive Oligopyrrole/Chitosan Hydrogel Intended for Cartilage Tissue Engineering. Int. J. Biol. Macromol..

[58] Vishwanath V., Pramanik K., Biswas A. (2016). Optimization and Evaluation of Silk Fibroin-Chitosan Freeze-Dried Porous Scaffolds for Cartilage Tissue Engineering Application. J. Biomater. Sci. Polym. Ed..

[59] Kar S., Kaur T., Thirugnanam A. (2016). Microwave-Assisted Synthesis of Porous Chitosan–Modified Montmorillonite–Hydroxyapatite Composite Scaffolds. Int. J. Biol. Macromol..

[60] Tithito T., Suntornsaratoon P., Charoenphandhu N., Thongbunchoo J., Krishnamra N., Tang I.M., Pon-On W. (2019). Fabrication of Biocomposite Scaffolds Made with Modified Hydroxyapatite Inclusion of Chitosan-Grafted-Poly(Methyl Methacrylate) for Bone Tissue Engineering. Biomed. Mater..

[61] Fiqrianti I., Widiyanti P., Manaf M., Savira C., Cahyani N., Bella F. (2018). Poly-L-Lactic Acid (PLLA)-Chitosan-Collagen Electrospun Tube for Vascular Graft Application. JFB.

[62] Pezeshki-Modaress M., Zandi M., Rajabi S. (2018). Tailoring the Gelatin/Chitosan Electrospun Scaffold for Application in Skin Tissue Engineering: An in Vitro Study. Prog. Biomater..

[63] Jafari A., Hassanajili S., Azarpira N., Bagher Karimi M., Geramizadeh B. (2019). Development of Thermal-Crosslinkable Chitosan/Maleic Terminated Polyethylene Glycol Hydrogels for Full Thickness Wound Healing: In Vitro and in Vivo Evaluation. Eur. Polym. J..

[64] Madni A., Khan R., Ikram M., Naz S.S., Khan T., Wahid F. (2019). Fabrication and Characterization of Chitosan–Vitamin C–Lactic Acid Composite Membrane for Potential Skin Tissue Engineering. Int. J. Polym. Sci..

[65] Zhang N., Gao T., Wang Y., Liu J., Zhang J., Yao R., Wu F. (2020). Modulating Cationicity of Chitosan Hydrogel to Prevent Hypertrophic Scar Formation during Wound Healing. Int. J. Biol. Macromol..

[66] Liu Y., Mao J., Guo Z., Hu Y., Wang S. (2022). Polyvinyl Alcohol/Carboxymethyl Chitosan Hydrogel Loaded with Silver Nanoparticles Exhibited Antibacterial and Self-Healing Properties. Int. J. Biol. Macromol..

[67] Chang G., Dang Q., Liu C., Wang X., Song H., Gao H., Sun H., Zhang B., Cha D. (2022). Carboxymethyl Chitosan and Carboxymethyl Cellulose Based Self-Healing Hydrogel for Accelerating Diabetic Wound Healing. Carbohydr. Polym..

[68] Mishra A.H., Mishra D. (2020). Evidences of Biomimetic and Nonantibiotic Characteristics of the Zinc–Carboxymethyl Chitosan–Genipin Organometallic Complex and Its Biocompatibility Aspects. Biomacromolecules.

[69] Liu J., Yang B., Li M., Li J., Wan Y. (2020). Enhanced Dual Network Hydrogels Consisting of Thiolated Chitosan and Silk Fibroin for Cartilage Tissue Engineering. Carbohydr. Polym..

[70] Janarthanan G., Tran H.N., Cha E., Lee C., Das D., Noh I. (2020). 3D Printable and Injectable Lactoferrin-Loaded Carboxymethyl Cellulose-Glycol Chitosan Hydrogels for Tissue Engineering Applications. Mater. Sci. Eng. C.

[71] Li T., Song X., Weng C., Wang X., Gu L., Gong X., Wei Q., Duan X., Yang L., Chen C. (2019). Silk Fibroin/Carboxymethyl Chitosan Hydrogel with Tunable Biomechanical Properties Has Application Potential as Cartilage Scaffold. Int. J. Biol. Macromol..

[72] Rui Q., Gao J., Yin Z.-Z., Li J., Cai W., Wu D., Kong Y. (2022). A Biodegradable PH and Glutathione Dual-Triggered Drug Delivery System Based on Mesoporous Silica, Carboxymethyl Chitosan and Oxidized Pullulan. Int. J. Biol. Macromol..

[73] Yao S., Chen S., Wang R., Zhang K., Lin X., Mai S. (2022). Antibacterial Activity and Bonding Performance of Carboxymethyl Chitosan–Containing Dental Adhesive System. Int. J. Adhes. Adhes..

[74] Yin H., Song P., Chen X., Huang Q., Huang H. (2022). A Self-Healing Hydrogel Based on Oxidized Microcrystalline Cellulose and Carboxymethyl Chitosan as Wound Dressing Material. Int. J. Biol. Macromol..

[75] Zhang X., Chen Y., Han J., Mo J., Dong P., Zhuo Y., Feng Y. (2019). Biocompatiable Silk Fibroin/Carboxymethyl Chitosan/Strontium Substituted Hydroxyapatite/Cellulose Nanocrystal Composite Scaffolds for Bone Tissue Engineering. Int. J. Biol. Macromol..

[76] Kashyap P.K., Chauhan S., Negi Y.S., Goel N.K., Rattan S. (2022). Biocompatible Carboxymethyl Chitosan-Modified Glass Ionomer Cement with Enhanced Mechanical and Anti-Bacterial Properties. Int. J. Biol. Macromol..

[77] Xu C., Guan S., Wang S., Gong W., Liu T., Ma X., Sun C. (2018). Biodegradable and Electroconductive Poly(3,4-Ethylenedioxythiophene)/Carboxymethyl Chitosan Hydrogels for Neural Tissue Engineering. Mater. Sci. Eng. C.

[78] Hao Y., Zhao W., Zhang H., Zheng W., Zhou Q. (2022). Carboxymethyl Chitosan-Based Hydrogels Containing Fibroblast Growth Factors for Triggering Diabetic Wound Healing. Carbohydr. Polym..

[79] Tao F., Cheng Y., Tao H., Jin L., Wan Z., Dai F., Xiang W., Deng H. (2020). Carboxymethyl Chitosan/Sodium Alginate-Based Micron-Fibers Fabricated by Emulsion Electrospinning for Periosteal Tissue Engineering. Mater. Des..

[80] Liu T., Feng Z., Li Z., Lin Z., Chen L., Li B., Chen Z., Wu Z., Zeng J., Zhang J. (2022). Carboxymethyl Chitosan/Sodium Alginate Hydrogels with Polydopamine Coatings as Promising Dressings for Eliminating Biofilm and Multidrug-Resistant Bacteria Induced Wound Healing. Int. J. Biol. Macromol..

[81] Osorio Echavarría J., Gómez Vanegas N.A., Orozco C.P.O. (2022). Chitosan/Carboxymethyl Cellulose Wound Dressings Supplemented with Biologically Synthesized Silver Nanoparticles from the Ligninolytic Fungus Anamorphous Bjerkandera Sp. R1. Heliyon.

[82] Maji S., Agarwal T., Das J., Maiti T.K. (2018). Development of Gelatin/Carboxymethyl Chitosan/Nano-Hydroxyapatite Composite 3D Macroporous Scaffold for Bone Tissue Engineering Applications. Carbohydr. Polym..

[83] Lu H.-T., Lu T.-W., Chen C.-H., Lu K.-Y., Mi F.-L. (2018). Development of Nanocomposite Scaffolds Based on Biomineralization of N,O-Carboxymethyl Chitosan/Fucoidan Conjugates for Bone Tissue Engineering. Int. J. Biol. Macromol..

[84] Wang J., Liu J., Lu D.-Q., Chen L., Yang R., Liu D., Zhang B. (2022). Diselenide-Crosslinked Carboxymethyl Chitosan Nanoparticles for Doxorubicin Delivery: Preparation and in Vivo Evaluation. Carbohydr. Polym..

[85] Medeiros Borsagli F.G.L., de Souza A.J.M., Paiva A.E. (2020). Ecofriendly Multifunctional Thiolated Carboxymethyl Chitosan-Based 3D Scaffolds with Luminescent Properties for Skin Repair and Theragnostic of Tissue Regeneration. Int. J. Biol. Macromol..

[86] Murugan E., Akshata C.R., Ilangovan R., Mohan M. (2022). Evaluation of Quaternization Effect on Chitosan-HAP Composite for Bone Tissue Engineering Application. Colloids Surf. B Biointerfaces.

[87] Zhou M., Liao J., Li G., Yu Z., Xie D., Zhou H., Wang F., Ren Y., Xu R., Dai Y. (2022). Expandable Carboxymethyl Chitosan/Cellulose Nanofiber Composite Sponge for Traumatic Hemostasis. Carbohydr. Polym..

[88] Yu N., Li Y., Wang Y., Xu H., Ye F., Fu Q. (2022). Healing Effect of Carboxymethyl Chitosan-Plantamajoside Hydrogel on Burn Wound Skin. Burns.

[89] Ghahremanzadeh F., Alihosseini F., Semnani D. (2021). Investigation and Comparison of New Galactosylation Methods on PCL/Chitosan Scaffolds for Enhanced Liver Tissue Engineering. Int. J. Biol. Macromol..

[90] Shaheen T.I., Abdelhameed M.F., Zaghloul S., Montaser A.S. (2022). In Vivo Assessment of the Durable, Green and in Situ Bio-Functional Cotton Fabrics Based Carboxymethyl Chitosan Nanohybrid for Wound Healing Application. Int. J. Biol. Macromol..

[91] Liu H., Mao J., Yao K., Yang G., Cui L., Cao Y. (2004). A Study on a Chitosan-Gelatin-Hyaluronic Acid Scaffold as Artificial Skin in Vitro and Its Tissue Engineering Applications. J. Biomater. Sci. Polym. Ed..

[92] Jin Z., Hu G., Zhao K. (2022). Mannose-Anchored Quaternized Chitosan/Thiolated Carboxymethyl Chitosan Composite NPs as Mucoadhesive Carrier for Drug Delivery. Carbohydr. Polym..

[93] Hasan A., Waibhaw G., Saxena V., Pandey L.M. (2018). Nano-Biocomposite Scaffolds of Chitosan, Carboxymethyl Cellulose and Silver Nanoparticle Modified Cellulose Nanowhiskers for Bone Tissue Engineering Applications. Int. J. Biol. Macromol..

[94] Cheng F., Xu L., Dai J., Yi X., He J., Li H. (2022). N, O-Carboxymethyl Chitosan/Oxidized Cellulose Composite Sponge Containing ε-Poly-l-Lysine as a Potential Wound Dressing for the Prevention and Treatment of Postoperative Adhesion. Int. J. Biol. Macromol..

[95] Zhang H., Gu Z., Li W., Guo L., Wang L., Guo L., Ma S., Han B., Chang J. (2022). PH-Sensitive O-Carboxymethyl Chitosan/Sodium Alginate Nanohydrogel for Enhanced Oral Delivery of Insulin. Int. J. Biol. Macromol..

[96] Sharifi F., Atyabi S.M., Norouzian D., Zandi M., Irani S., Bakhshi H. (2018). Polycaprolactone/Carboxymethyl Chitosan Nanofibrous Scaffolds for Bone Tissue Engineering Application. Int. J. Biol. Macromol..

[97] Jiang Z., Li L., Li H., Xia L., Hu H., Wang S., Liu C., Chi J., Yang Y., Song F. (2022). Preparation, Biocompatibility, and Wound Healing Effects of O-Carboxymethyl Chitosan Nonwoven Fabrics in Partial-Thickness Burn Model. Carbohydr. Polym..

[98] Yang Y., Campbell Ritchie A., Everitt N.M. (2021). Recombinant Human Collagen/Chitosan-Based Soft Hydrogels as Biomaterials for Soft Tissue Engineering. Mater. Sci. Eng. C.

[99] Shakir M., Jolly R., Khan M.S., Iram N., Sharma T.K., Al-Resayes S.I. (2015). Synthesis and Characterization of a Nano-Hydroxyapatite/Chitosan/Polyethylene Glycol Nanocomposite for Bone Tissue Engineering: Nano-hydroxyapatite/chitosan/polyethylene glycol nanocomposite. Polym. Adv. Technol..

[100] Chi J., Jiang Z., Chen X., Peng Y., Liu W., Han B., Han B. (2019). Studies on Anti-Hepatocarcinoma Effect, Pharmacokinetics and Tissue Distribution of Carboxymethyl Chitosan Based Norcantharidin Conjugates. Carbohydr. Polym..

[101] Rao K.M., Sudhakar K., Suneetha M., Won S.Y., Han S.S. (2021). Fungal-Derived Carboxymethyl Chitosan Blended with Polyvinyl Alcohol as Membranes for Wound Dressings. Int. J. Biol. Macromol..

[102] Xie M., Zeng Y., Wu H., Wang S., Zhao J. (2022). Multifunctional Carboxymethyl Chitosan/Oxidized Dextran/Sodium Alginate Hydrogels as Dressing for Hemostasis and Closure of Infected Wounds. Int. J. Biol. Macromol..

[103] Yang L., Lan Y., Guo H., Cheng L., Fan J., Cai X., Zhang L., Chen R., Zhou H. (2010). Ophthalmic Drug-Loaded N,O-Carboxymethyl Chitosan Hydrogels: Synthesis, in Vitro and in Vivo Evaluation. Acta Pharm. Sin..

